# The Decrease in Serum Total Cholesterol and Low-Density Lipoprotein (LDL) Concentrations With the Initiation of Hemodialysis Despite a Concomitant Increase in Serum Albumin Concentrations

**DOI:** 10.7759/cureus.47272

**Published:** 2023-10-18

**Authors:** Ramin Sam, Li Zhang, Delphine S Tuot, Rafia Chaudhry

**Affiliations:** 1 Nephrology, Zuckerberg San Francisco General Hospital, University of California, San Francisco, San Francisco, USA; 2 Epidemiology and Biostatistics, University of California, San Francisco, San Francisco, USA; 3 Medicine/Nephrology, Zuckerberg San Francisco General Hospital, University of California, San Francisco, San Francisco, USA

**Keywords:** cholesterol, lipid panel, malnutrition, albumin, hemodialysis

## Abstract

Background and objective

Hemodialysis patients often have lower serum low-density lipoprotein (LDL) and total cholesterol concentrations compared to the general population. It is unclear if this is due to a persistent decline in the values due to kidney disease or if the hemodialysis itself is contributing to the lower values. It is often assumed that malnutrition and anorexia are the main causes of the low lipid concentration in hemodialysis patients. In this study, we aimed to determine the association between hemodialysis initiation and serum lipid and albumin concentrations.

Methodology

The medical records of all patients initiating hemodialysis over an 11-year period at a single center were retrospectively reviewed. The data of 145 patients who had all the required lab values available were ultimately included in the study. Serum lipid levels at the initiation of hemodialysis were compared to values obtained mostly 6-18 months later. In order to determine if poor nutritional status is the reason for the decline in serum lipid levels, the serum albumin concentration at the initiation of hemodialysis was compared to that obtained during follow-up labs.

Results

We observed that serum cholesterol concentration declined from an average of 147 mg/dL to 137 mg/dL, while LDL decreased from an average of 78 mg/dL to 68 mg/dL, and serum albumin concentration increased from 3.4 g/dL to 3.8 g/dL after an average follow-up period of 10.8 months.

Conclusions

Based on our findings, the decline in serum LDL and total cholesterol concentrations with the initiation of hemodialysis may not be attributed to poor nutritional intake.

## Introduction

In the 1990s, several studies suggested that the initiation of hemodialysis led to lower serum lipid concentrations, specifically lower serum total cholesterol and low-density lipoprotein (LDL) levels [1-9]. However, the findings of these small case series were not consistently reproduced in later studies [10-12]. More recently, studies have suggested that patients with advanced chronic kidney disease (CKD) not yet on dialysis and those with end-stage renal disease (ESRD) on dialysis have both been observed to have decreased serum total cholesterol, LDL, and high-density lipoprotein (HDL) levels compared to the general population, whereas total triglyceride concentrations have been high in CKD and low in ESRD patients [13]. The association between kidney disease severity and serum lipid concentrations is an important area of research given the high cardiovascular morbidity as well as the recent data showing no benefit from lipid-lowering therapy on cardiovascular health in the hemodialysis population [14-16]. A better understanding of the relationship could help elucidate more optimal cardiovascular management strategies.

The mechanism underlying the association between ESRD and low lipid concentrations is not clear. Serum cholesterol with a molecular weight of 387 g/mole is fairly well dialyzable if it existed in serum as free cholesterol. However, fat molecules including cholesterol are extremely insoluble in plasma and are carried in the plasma only in the form of high-density and low-density lipoproteins. Large lipoprotein entities with molecular weights ranging from 250,000 to 3,000,000 g/mole are clearly not dialyzable, leading to the conclusion that lipid molecules cannot be directly removed from the circulation by hemodialysis. However, the above conclusion does not necessarily indicate that hemodialysis as a technique cannot lead to lower serum lipid concentration, as the metabolism of these molecules is complicated and depends on many factors.

Another possible cause of low lipid concentrations among individuals with ESKD is that with advancing CKD and an elevated uremic milieu, there is more malnutrition/inflammation that can lead to lower serum lipid concentrations over time. However, in this context, one would expect the serum albumin levels (a great indicator of nutritional status/inflammation) to decline concomitantly with the decline in serum lipids [17,18]. To explore this hypothesis, we reviewed the data of all patients starting hemodialysis at a single center over several years to determine the impact of hemodialysis initiation on serum lipid and albumin concentrations.

## Materials and methods

Study design and population

We conducted a retrospective study of all incident hemodialysis patients at a single center from January 2007 through December 2017. Out of the 174 patients identified, 29 did not have serum cholesterol or albumin concentration documented within two months of starting dialysis or after six months of starting hemodialysis and were thus excluded from the analysis. Hence, the final study population consisted of 145 incident hemodialysis patients.

Outcome measures

The primary outcome was the change in total serum cholesterol before versus after hemodialysis initiation. The change was calculated by using the serum cholesterol value within two months after the initiation of hemodialysis and the value after 6-33 months after the initiation of hemodialysis. Most patients had a follow-up level available within 6-12 months after initiation of hemodialysis (n=114/145; 79%). Secondary outcomes included changes in LDL cholesterol and serum albumin.

Of the 145 total patients studied, 129 had stable statin use over time, including 62 who were not receiving statins at the initiation of hemodialysis and were not started on one subsequently and 67 patients who were taking statins at the start of hemodialysis and continued until follow-up. Six patients were taking statins at the initiation of hemodialysis but had stopped it by the time of the follow-up labs and, finally, 10 patients were not receiving statin at the start of hemodialysis but were taking it by the time of follow-up labs.

Among the 145 patients, 80 did not initiate or increase the dose of sevelamer from the initiation of hemodialysis to the time of the follow-up labs and were thus deemed to be on a stable prescription. The average duration between initial labs and follow-up labs in this group of patients was 8.4 months. These patients were studied separately to determine the effects of sevelamer intake on changes in serum lipids.

Statistical analysis

Demographic and clinical characteristics of the study population were summarized via descriptive statistics. In general, frequency distribution and percentage were used to summarize categorical variables, and mean with standard deviation was used to describe continuous variables. Changes in absolute levels of serum lipids, albumin glucose, and glycosylated hemoglobin were calculated. Comparison of the continuous variables (serum lipid, albumin, glucose, glycosylated hemoglobin) between two different time points was assessed using the paired t-test. Subgroup analyses were performed among patients who had an initial serum LDL greater than 100 mg/dL (n=24) and those who did not initiate or increase the dose of sevelamer after dialysis initiation (n=80). The data were analyzed using Microsoft Excel (Microsoft Corp., Redmond, WA) and the statistical software R (https://www.r-project.org). A p-value <0.05 was considered statistically significant.

This study was approved by the local ethics committee (institutional review board) with approval #21-33581 and the need for informed consent was waived.

## Results

The patient population consisted of 145 individuals, with a mean age of 53.5 years (SD=13.3); 66% of them were male. Baseline characteristics of the cohort are shown in Tables 1-2.

**Table 1:** Baseline characteristics of the study population LDL: low-density lipoprotein; NA: not available

Variables		Overall	Patients with serum LDL >100 mg/dL
Number		145	24
Gender, n (%)	F	49 (33.8)	5 (20.8)
	M	96 (66.2)	19 (79.2)
Statin, n (%)	No	72 (49.7)	13 (54.2)
	Yes	73 (50.3)	11 (45.8)
Sevelamer, n (%)	No	73 (50.3)	20 (83.3)
	Yes	28 (19.3)	4 (16.7)
	NA	44 (30.3)	0

**Table 2:** Baseline characteristics of the study population (continued) HDL: high-density lipoprotein; LDL: low-density lipoprotein; SD: standard deviation

Variables	Overall, mean (SD)	Patients with serum LDL >100 mg/dL, mean (SD)
Age, years	53.49 (13.32)	48.41 (12.40)
Cholesterol, mg/dL	146.96 (45.97)	206.88 (44.70)
LDL, mg/dL	78.22 (43.47)	133.71 (42.60)
HDL, mg/dL	41.29 (14.90)	40.38 (10.50)
Triglycerides, mg/dL	163.65 (108.55)	164.67 (69.88)
Albumin, g/dL	3.35 (0.62)	3.10 (0.70)
Hemoglobin A1C, %	7.48 (1.84)	8.90 (2.63)
Glucose, mg/dL	147.24 (90.91)	135.75 (62.77)

**Figure 1:**
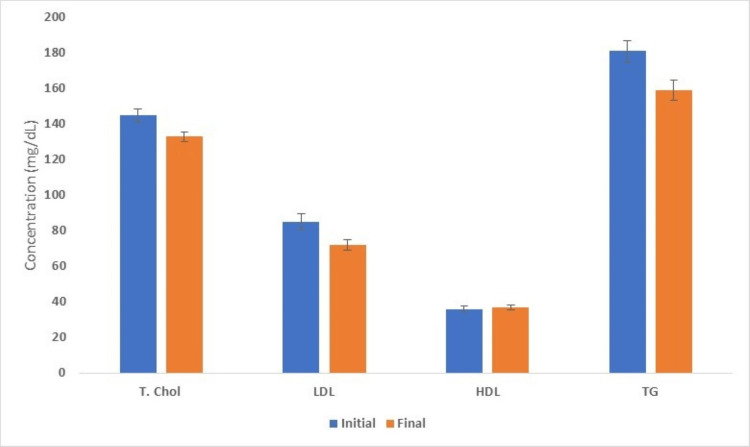
Serum lipid panel at the initiation of dialysis and after a few months HDL: high-density lipoprotein; LDL: low-density lipoprotein; T. Chol: total cholesterol; TG: triglycerides

The average initial total serum cholesterol concentration was 146.96 mg/dL ± 3.77 (standard error of the mean) (Figure 1). After an average of 10.8 months of hemodialysis, the serum total cholesterol concentration dropped to 136.98 ± 2.83 mg/dL. Initial LDL, HDL, and triglycerides levels were as follows: 78.22 ± 4.70, 41.29 ± 1.74, and 163.65 ± 6.1 mg/dL; final LDL, HDL, and triglycerides levels were as follows: 67.52 ± 2.94, 41.29 ± 1.40, and 150.44 ± 5.84 mg/dL. These data demonstrated an approximate 10 mg/dL drop in the serum total cholesterol, LDL, and serum triglyceride concentrations, while the serum HDL level remained stable with the initiation of hemodialysis (Table 3).

**Table 3:** Changes in serum laboratory values after the initiation of hemodialysis (N=145) HDL: high-density lipoprotein; LDL: low-density lipoprotein; SD: standard deviation

Variables	Values, mean (SD)
Hemoglobin A1C, %	0.33 (1.84)
Albumin, g/dL	0.45 (0.51)
Cholesterol, mg/dL	-9.98 (37.93)
Glucose, mg/dL	-2.61 (86.08)
HDL, mg/dL	1.64 (12.43)
LDL, mg/dL	-10.70 (34.32)
Triglycerides, mg/dL	-13.21 (87.04)
Weight, kg	0 (6.53)

**Figure 2:**
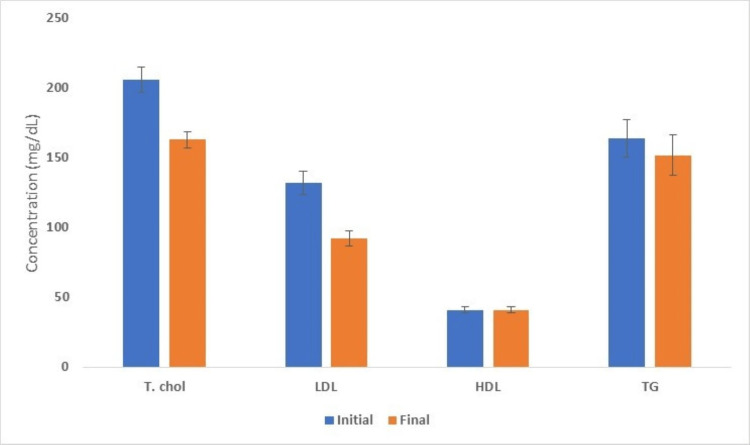
Serum lipid panel at the initiation of hemodialysis and after a few months in the subgroup of patients with serum LDL >100 mg/dL HDL: high-density lipoprotein; LDL: low-density lipoprotein; T. Chol: total cholesterol; TG: triglycerides

A subgroup of patients who had a serum LDL concentration >100 mg/dL at the initiation of hemodialysis were analyzed separately in order to gain deeper insights into the effect of hemodialysis on serum lipids (Tables 4-5). The initial average serum total cholesterol concentration in this group of 24 patients was 206.88 ± 8.84 mg/dL. The corresponding values for LDL, HDL, and triglyceride concentrations were 133.71 ± 8.45 mg/dL, 40.38 ± 2.06 mg/dL, and 164.67 ± 13.68 mg/dL. After an average of 8.8 months on dialysis, the average serum total cholesterol concentration, LDL, HDL, and triglyceride levels were 164.05 ± 5.75 mg/dL, 92.54 ± 5.36 mg/dL, 40.88 ± 2.09 mg/dL and 153.21 ± 14.74 mg/dL. The changes here were more pronounced with serum LDL, dropping by an average of more than 40 mg/dL (Figure 2).

**Table 4:** Baseline characteristics (subgroup with serum LDL >100 mg/dL, n=24) LDL: low-density lipoprotein

Variables		Values, n (%)
Gender	F	5 (20.8)
	M	19 (79.2)
Statin	No	13 (54.2)
	Yes	11 (45.8)
Sevelamer	No	20 (83.3)
	Yes	4 (16.7)

**Table 5:** Baseline characteristics (continued; subgroup with serum LDL >100 mg/dL, n=24) HDL: high-density lipoprotein; LDL: low-density lipoprotein; SD: standard deviation

Variables	Values, mean (SD)
Age, years	48.41 (12.40)
Cholesterol, mg/dL	206.88 (44.70)
LDL, mg/dL	133.71 (42.60)
HDL, mg/dL	40.38 (10.50)
Triglycerides, mg/dL	164.67 (69.88)
Albumin, g/dL	3.10 (0.70)
Hemoglobin A1C, %	8.90 (2.63)
Glucose, mg/dL	135.75 (62.77)

**Figure 3:**
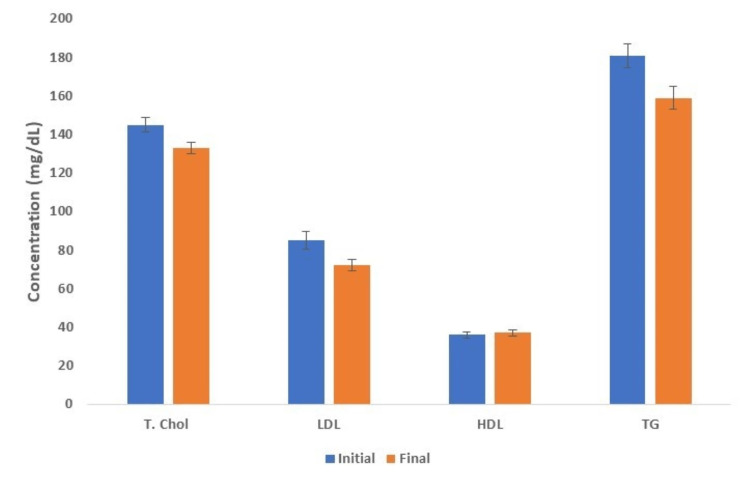
Serum lipid panel at the initiation of hemodialysis and after a few months after excluding patients who were not taking statin at the initiation of hemodialysis but taking them at the time of follow-up labs HDL: high-density lipoprotein; LDL: low-density lipoprotein; T. Chol: total cholesterol; TG: triglycerides

Among the 145 patients studied, only 10 were not receiving statin at the start of hemodialysis but were taking it by the time of follow-up. Excluding data from those 10 individuals, the mean total serum cholesterol decreased from 142.67 ± 3.77 mg/dL to 132.17 ± 2.83 mg/dL and the mean LDL decreased from 86.5 ± 4.7 mg/dL to 72.27 ± 2.94 mg/dL with the initiation of hemodialysis (Figure 3). This change represented a significant decrease in serum lipids after a few months of hemodialysis.

**Figure 4:**
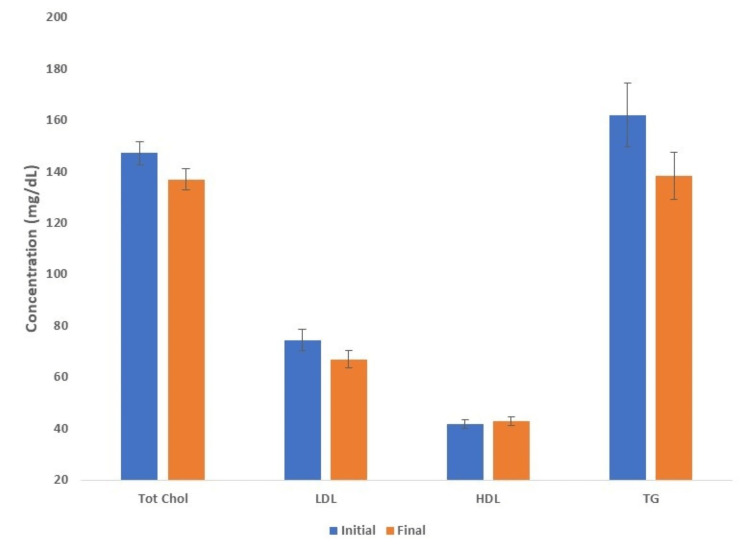
Serum lipid panel at the initiation of hemodialysis and after a few months after excluding patients who initiated or increased the dose of sevelamer between the initial and final labs HDL: high-density lipoprotein; LDL: low-density lipoprotein; T. Chol: total cholesterol; TG: triglycerides

Among patients with stable sevelamer prescription over time (n=80), the average initial serum total cholesterol, LDL, HDL, and triglycerides concentrations were 147.2 ± 4.6 mg/dL, 74.4 ± 4.2 mg/dL, 41.6 ± 1.7 mg/dL, and 162.1 ± 12.5 mg/dL, and that at the time of the follow-up labs were as follows: 137.0 ± 4.0 mg/dL, 66.9 ± 3.3 mg/dL, 43 ± 1.7 mg/dL, and 138.3 ± 9.1 mg/dL (Figure 4). The degree of the total cholesterol reduction was similar among the patients not started on sevelamer and the entire group (around -10 mg/dL), while the LDL declined by -7.5 mg/dL in the patients not initiated on sevelamer and by -10.7 in the entire group of patients. Hence, the decline in serum LDL was about 43% more in the entire group of patients compared to the group that did not have an increase in sevelamer dosing.

The average serum albumin concentration at the initiation of dialysis was 3.35 ± 0.06 g/dL, and after an average of 10.8 months, it increased to 3.80 ± 0.04 (Table 3). Among stable sevelamer users, albumin concentrations changed from a mean of 3.2 ± 0.07 g/dL to 3.7 ± 0.05 g/dL. The average random serum glucose concentration at the initiation of hemodialysis was 147.24 ± 7.55 mg/dL, and after an average of 10.8 months on hemodialysis, it was 144.63 ± 7.09 mg/dL (Table 3). The corresponding values for the serum hemoglobin A1C were 7.48% ± 0.28 and 7.81% ± 0.32. Despite no apparent change in the serum glucose concentration, there appeared to be an increase in the hemoglobin A1C with improved nutritional status of the patients after a few months on dialysis.

Patients’ weight was also examined both at the time of the initial labs and at the time of the final labs. The average patient weight at the time of the initial labs was 75.3 kg and it was the same at the time of the final labs. The average change in weight was 0 ± 0.54 kg. In total, 71 patients gained weight, 56 patients lost weight, two patients' weight remained exactly the same, and 16 patients did not have weight data available.

Changes in LDL, albumin, and glucose concentrations were analyzed by using the paired t-test. There was a statistically significant increase in serum albumin, a decrease in serum LDL, and no significant change in serum glucose (Table 6). The same method (in addition to the Wilcoxon rank-sum test) was also used among the subgroup of patients with serum LDL >100 mg/dL (Tables 7-8).

**Table 6:** Change in serum albumin, LDL, and glucose between the initiation of hemodialysis and after a few months (range: 8.4-10.8 months) LDL: low-density lipoprotein; SD: standard deviation

Variables	Overall (n=145)			Patients with serum LDL >100 mg/dL (n=24)		
	Initial, mean (SD)	Final, mean (SD)	P-value	Initial, mean (SD)	Final, mean (SD)	P-value
LDL, mg/dL	78.22 (43.47)	67.52 (28.82)	0.003	133.71 (42.60)	92.54 (27.10)	<0.001
Albumin, g/dL	3.35 (0.62)	3.81 (0.45)	<0.001	3.10 (0.70)	3.85 (0.44)	<0.001
Glucose, mg/dL	147.24 (90.91)	144.63 (85.37)	0.6341	135.75 (62.77)	156.50 (96.93)	0.6341

**Table 7:** Significance of the change in serum albumin, LDL, and glucose between the initiation of hemodialysis and after a few months in the subgroup of patients with serum LDL >100 mg/dL based on t-test LDL: low-density lipoprotein; SD: standard deviation

Variables	Initial, mean (SD)	Final, mean (SD)	P-value
LDL, mg/dL	133.71 (42.60)	92.54 (27.10)	<0.001
Albumin, g/dL	3.10 (0.70)	3.85 (0.44)	<0.001
Glucose, mg/dL	135.75 (62.77)	156.50 (96.93)	0.6341

**Table 8:** Significance of the change in serum albumin, LDL, and glucose between the initiation of hemodialysis and after a few months in the subgroup of patients with serum LDL >100 mg/dL based on Wilcoxon rank-sum test LDL: low-density lipoprotein; IQR: interquartile range

Variables	Initial, median (IQR)	Final, median (IQR)	P-value
LDL, mg/dL	121.00 (111.75, 135.25)	92.00 (81.75, 104.75)	<0.001
Albumin, g/dL	3.00 (2.60, 3.68)	3.95 (3.60, 4.12)	<0.001
Glucose, mg/dL	115.50 (86.75, 161.50)	120.00 (88.00, 193.50)	0.966

## Discussion

In this single-center study involving incident hemodialysis patients, we found that serum total cholesterol and LDL cholesterol decreased after hemodialysis initiation while levels of serum albumin increased. These data are consistent with that of a few prior studies examining the impact of hemodialysis on serum cholesterol levels. In 1982, Chan et al. analyzed 76 patients on hemodialysis and compared them to 26 patients on peritoneal dialysis and found that the serum total cholesterol concentration was significantly lower in patients on hemodialysis compared to controls and patients on peritoneal dialysis [1]. In 1995, Hörkkö et al. assessed 11 patients who had been on hemodialysis, nine patients on peritoneal dialysis, and nine controls [2]. They found the average serum total cholesterol to be 4.52 mmol/L (175 mg/dL) in hemodialysis patients, 6.59 mmol/L (255 mg/dL) in peritoneal dialysis patients, and 4.82 mmol/L (187 mg/dL) in controls. The corresponding values for the serum LDL concentrations were 2.26 mmol/L (87 mg/dL), 3.33 mmol/L (129 mg/dL), and 2.66 mmol/L (103 mg/dL). In 2016, Lokesh et al. compared the serum lipid panels in 40 patients on hemodialysis with 40 age- and sex-matched controls, and they found the average LDL concentration to be 71.4 mg/dL in the hemodialysis patients and 102.5 mg/dL in the controls [13].

Several studies have also tried to investigate whether increasing the hemodialysis dose or the flux of the dialyzer would lead to lower serum total cholesterol and LDL concentrations. Seres et al. measured serum lipid profile pre- and post-dialysis in nine patients undergoing hemodialysis with a cellulose ester dialyzer and compared them to 10 patients dialyzing with a polysulfone high flux dialyzer [3]. They found a significant decrease associated with hemodialysis in triglyceride concentrations in patients undergoing hemodialysis with a polysulfone dialyzer, while no change in patients hemodialyzing with cellulose dialyzers was seen. It was hypothesized that a circulating substance would inhibit lipoprotein lipase in uremic patients, which is removed by high flux polysulfone dialyzer but not with cellulose dialyzers. de Précigout et al. switched seven patients (who had been on dialysis for an average of 41 months) from cellulose-based dialyzer to polyamide membrane hemodialysis (4). The patients received hemodialysis with polyamide dialyzers for four consecutive months. The serum total cholesterol concentration dropped from 6.07 mmol/L (235 mg/dL) to 5.60 mmol/L (217 mg/dL) at the end of the fourth month. The authors hypothesized that high flux hemodialysis enables the removal of circulating inhibitors of lipoprotein lipase, either of apolipoproteins (such as apolipoprotein C3) or of an unknown substance. In the largest study on this topic so far, Azak et al. randomized 312 patients who were already on hemodialysis for an average of five years to high flux or low flux dialyzers [5]. After six months of follow-up, the serum LDL decreased by 9.7% and the serum total cholesterol decreased by 9.4% in the high flux group, whereas those values increased in the low flux dialyzer group.

Not all studies involving high flux dialyzers have found decreased lipid concentrations with the use of these dialyzers. Wei et al. switched regular hemodialysis to hemodiafiltration in six patients on hemodialysis for a mean period of 7.1 years [10]. After four weeks of hemodiafiltration, the serum total cholesterol increased from 157 mg/dL to 172 mg/dL and the serum LDL increased from 79 mg/dL to 106 mg/dL which was not statistically significant. A study of 42 patients who were switched from cellulosic dialysis membrane to polyacrylonitrile dialyzers found no change in the lipid panel after 12 weeks of change in treatment [11]. Another study where 45 patients were randomized to low and high flux dialyzers showed no difference in total serum cholesterol or serum LDL levels [12]. Even though the findings of these experiments have been inconsistent, most of the evidence shows a likely lowering of serum total cholesterol and LDL concentration with the initiation of hemodialysis or an increase in the dose or the flux of the hemodialysis. Our findings were consistent with these historical data: we noted an average decrease of 10 mg/dL in serum total cholesterol and LDL concentration after a few months of initiating hemodialysis. The decrease in serum lipids was much more pronounced in patients who started hemodialysis with an elevated lipid panel. Combining all available data, it is likely that initiating hemodialysis does lead to lower serum total cholesterol and LDL concentration. The next question that needs to be addressed is what mediates this effect.

Does worsening nutritional status explain the lowering of serum lipids with the initiation of hemodialysis?

One possibility is that the decrease in serum lipids is due to poor nutritional intake or high inflammatory state, as hemodialysis patients are often ill and malnourished. To exclude this possibility, serum albumin concentration was analyzed pre- and post-initiation of hemodialysis at the same time as serum lipid levels. Despite the significant drop in serum lipid levels, the serum albumin actually increased with the initiation of hemodialysis, consistent with the observation that uremic patients feel and eat better after starting hemodialysis. The above observation almost entirely rules out the possibility that the drop in serum lipids is mediated by poor nutritional intake or a higher inflammatory state.

Is the increase in serum albumin with the initiation of hemodialysis a result of more fluid removal?

In order to exclude this possibility, the weight of the patients at the initiation of hemodialysis was compared to their weight at the time of the final labs. It was found that the weights did not change and, in fact, more patients gained weight than lost weight. Thus, it seems unlikely that the increase in serum albumin is due to fluid loss.

Does statin or sevelamer initiation or up-titration explain the drop in serum lipids?

Another possible cause for the drop in lipid parameters with the initiation of hemodialysis is closer follow-up care and more aggressive use of cholesterol-lowering medications. To exclude this possibility, it was found that only 10 of the 145 patients initiated a statin during the follow-up period and if one were to exclude these patients, the results of the study would not be affected. Thus, it does not seem that statin therapy explains the drop in lipid parameters with the initiation of hemodialysis. Similarly, there are well-controlled trials that showed sevelamer therapy reduces serum lipid levels [19]. It is safe to assume that sevelamer use will increase upon initiation of hemodialysis. To determine the size of this effect, all the patients who initiated sevelamer or increased the dose of the sevelamer were excluded from the sub-analysis. Among the 80 patients in this subgroup, the LDL concentration dropped from 74.4 mg/dL to 66.9 mg/dL compared to a lowering of LDL from 78.2 mg/dL to 67.5 mg/dL in the entire cohort. Percentagewise, the drop in serum LDL was 10% in the group that did not have increased use of sevelamer compared to 14% in the entire group of patients. The conclusion from this sub-analysis is that sevelamer does likely explain some of the drop in serum LDL with the initiation of hemodialysis but not all of it. 

Is better glycemic control the reason behind improved lipid panels with the initiation of hemodialysis?

Clearly, better sugar control in diabetic patients can result in lower triglyceride levels. However, serum LDL concentration is likely not affected [20]. With worsening renal function, there is clearly a reduction in glycosylated hemoglobin concentration probably due to decreased gluconeogenesis by the kidneys. To exclude any role of worsening renal function with the initiation of dialysis on changes in the lipid panel with the initiation of hemodialysis, glycosylated hemoglobin concentration was analyzed. There was an actual increase in serum glycosylated hemoglobin concentration with the initiation of dialysis, which is in agreement with the increased serum albumin concentration. Any role of better diabetes control in improving lipid parameters is thus excluded. 

Is it possible the kidneys are a lipidogenic organ similar to being a gluconeogenic organ?

The next possibility is that the kidneys produce lipids and, with worsening renal function, the amount of lipid production from the kidneys drops and the serum LDL and total cholesterol concentration are reduced. One study showed the kidneys were the major site of circulating mevalonic acid uptake and metabolism [21]. Mevalonic acid is subsequently metabolized to cholesterol. The fact that serum LDL levels are low often prior to the initiation of hemodialysis can be used to argue that the kidneys can truly produce lipids; however, an alternative explanation is the poor nutritional status of uremic patients immediately prior to starting dialysis. However, the latter theory does not explain why the serum lipid concentrations continue to drop with the initiation of dialysis despite improving nutritional markers. We cannot entirely exclude the possibility of kidneys being a lipidogenic organ in terms of causing or contributing to low LDL and total cholesterol levels in patients who have recently started hemodialysis.

Does hemodialysis somehow lead to the lowering of serum LDL and serum total cholesterol concentration?

Finally, the last possibility is that the hemodialysis itself, through the removal of an unknown substance, will lead to the lowering of serum LDL and serum total cholesterol concentration. At this time, we believe this is the most likely possibility, although, clearly, the initiation of sevelamer therapy also plays a major role.

What are the implications of lowering LDL and total cholesterol concentration with the initiation of hemodialysis?

In 2005, the 4D trial randomized 1255 dialysis patients with type 2 diabetes to 20 mg of atorvastatin daily or matching placebo [22]. At baseline, the serum LDL was 121 mg/dL in the atorvastatin group and 125 mg/dL in the placebo group. With the initiation of atorvastatin, the serum LDL decreased by 42% in the atorvastatin group and 1.3% in the placebo group after four weeks. After a median follow-up of almost four years, there was no statistically significant difference in mortality or cardiovascular outcomes between the two groups. However, it seemed that the risk of stroke was higher and the risk of cardiac disease lower in the atorvastatin group. Interestingly, by the time of the conclusion of the study, the serum LDL concentration was around 100 mg/dL in the placebo group and 70 mg/dL in the atorvastatin group.

The above-mentioned results were confirmed a few years later by the AURORA Study [14]. The AURORA trial randomized 2776 patients on hemodialysis to rosuvastatin 10 mg daily or matching placebo. After three months of follow-up, the serum LDL decreased by 43% in the rosuvastatin group compared to 1.9% in the placebo group. The median follow-up was 3.8 years in all patients, and there was no significant difference in outcomes including mortality, non-fatal myocardial infarction, or strokes between the two groups. The serum LDL concentration averaged 100 mg/dL in the rosuvastatin group and 99 mg/dL in the placebo group at the start of the study but was closer to 90 mg/dL in the placebo group and 60 mg/dL in the rosuvastatin group by the end of the study.

Another large trial to study statin therapy in patients with CKD and patients on dialysis was the SHARP study [6]. Published in 2011, the SHARP study randomized 9270 patients with CKD, including 3023 on dialysis, to receive simvastatin 20 mg daily in addition to ezetimibe 10 mg daily versus matching placebo. The baseline LDL levels were 107 mg/dL in the simvastatin group, 108 mg/dL in the placebo group, and 100 mg/dL in the entire cohort of patients on dialysis. The median follow-up period was 4.9 years for all the patients. With therapy, the serum LDL decreased by 33 mg/dL in the entire cohort and 23 mg/dL in the dialysis subgroup of the intervention groups. There was a 17% decrease in the first major atherosclerotic event (non-fatal myocardial infarction, coronary death, stroke, or peripheral artery disease procedure) in the intervention group compared to the placebo arm, and being on dialysis did not affect this outcome. In total, there were 1533 patients on dialysis (1275 on hemodialysis) in the placebo group and 1490 patients on dialysis (1252 patients on hemodialysis) in the simvastatin plus ezetimibe arm. It is important to note that statins should be continued in hemodialysis patients if indicated.

In order to put the above results into perspective, one needs to compare these results with those obtained in patients without kidney disease. The 4S study published in 1994 was likely the first large study to look into the effects of statins on cardiovascular outcomes [23]. Randomizing 4444 patients with coronary disease to simvastatin 20-40 mg daily or matching placebo, the 4S study followed patients for an average of 5.4 years. The baseline serum cholesterol concentration was 213-310 mg/dL in the entire cohort. Following statin therapy, serum cholesterol decreased by 25-35% and serum LDL by 38%. Cardiovascular events occurred in 28% of the placebo group and 19% of the simvastatin group (a 34% reduction in events). Mortality in the simvastatin group was 70% of the mortality in the placebo group, with the mortality rate in the entire cohort being close to 10%. By contrast, mortality in the AURORA study was 46% of that in the atorvastatin group and 48% of the mortality in the placebo group, which did not show statistical significance. However, when comparing these studies, one has to keep several factors in mind. Firstly, the initial cholesterol and LDL concentrations were much higher in the 4S study than in the AURORA study. Also, hemodialysis patients have a higher mortality rate due to their underlying disease compared to stable patients with coronary disease and a large part of this mortality may not be due to ischemic heart disease.

A more relevant study for our purposes would involve comparing patients with an LDL of around 100 mg/dL (the baseline LDL in ESRD patients) to patients with an LDL of around 70 mg/dL (the LDL in ESRD patients after treatment with statins). In 2004, the PROVE IT-TIMI 22 study was published, which compared 40 mg of pravastatin to 80 mg of atorvastatin in 4162 presenting with acute coronary syndrome [24]. The serum LDL dropped to an average of 95 mg/dL with pravastatin treatment and an average of 62 mg/dL with atorvastatin. After 18-36 months of follow-up, the primary outcome of death, myocardial infarction, stroke, revascularization, and rehospitalization of unstable angina was seen in 26.3% of the patients in the pravastatin group and 22.4% of the patients in the atorvastatin group, which indicated a clear, significant 16% advantage in the lower LDL group. This benefit seen here was about half the benefit seen in the 4S study. After this study, the guideline-recommended goal for LDL in patients with known coronary disease was lowered from less than 100 mg/dL to less than around 70 mg/dL. The mortality rate was low in this study: 2.2% in the atorvastatin group and 3.2% in the pravastatin group, and the difference did not achieve statistical significance. However, clearly, this study cannot be compared to the studies on dialysis patients, nor to those with a much higher mortality rate of 45-50%. The high mortality rate in dialysis patients, even though correctly attributed to mostly cardiac causes, may not all be amenable to coronary risk reductions (a large part of the mortality may be the result of severe LVH from years of hypertension leading to arrhythmia, arrhythmias due to electrolyte imbalances/shifts, fluid shifts leading to blood pressure instability, etc.).

Putting all these data together, the best conclusion would be that the benefit of cholesterol reduction in hemodialysis patients is not as high as in patients with coronary disease and elevated cholesterol levels, mainly due to the presence of low LDL levels in hemodialysis patients even without therapy. Secondly, hemodialysis patients have a much higher mortality rate than patients with coronary disease alone and many of the factors leading to such high mortality may not be amenable to coronary risk reduction, making it more difficult to establish benefits in hemodialysis patients.

This study has a few limitations. Not all patients had documented data for all of the values analyzed; moreover, we did not have a lot of patients with serum LDL concentrations over 100 mg/dL. Hence, we recommend further studies to gain deeper insights into the cause of the decline in serum lipid levels with the initiation of hemodialysis.

## Conclusions

Hemodialysis patients have low serum LDL and total cholesterol concentrations, and the initiation of dialysis leads to further lowering of these lipid parameters despite improving nutritional status. This could be attributed to either a direct/indirect effect of hemodialysis in lowering serum lipid levels or the possibility that the kidneys actually produce lipids. Additionally, some of the effects are due to the increase in sevelamer use with hemodialysis initiation. The already low and decreasing LDL levels in hemodialysis patients are likely the major cause for the lack of benefits of statin therapy observed in recent trials.

## References

[1] Chan MK, Varghese Z, Persaud JW, Baillod RA, Moorhead JF (1982). Hyperlipidemia in patients on maintenance hemo- and peritoneal dialysis: the relative pathogenetic roles of triglyceride production and triglyceride removal. Clin Nephrol.

[2] Hörkkö S, Huttunen K, Kesäniemi YA (1995). Decreased clearance of low-density lipoprotein in uremic patients under dialysis treatment. Kidney Int.

[3] Seres DS, Strain GW, Hashim SA, Goldberg IJ, Levin NW (1993). Improvement of plasma lipoprotein profiles during high-flux dialysis. J Am Soc Nephrol.

[4] de Précigout V, Higueret D, Larroumet N (1996). Improvement in lipid profiles and triglyceride removal in patients on polyamide membrane hemodialysis. Blood Purif.

[5] Azak A, Huddam B, Öneç K, Koçak G, Dede F, Aylı D, Duranay M (2012). Contribution of high flux membranes to the therapy of uremia-associated dyslipidemia. Ther Apher Dial.

[6] Baigent C, Landray MJ, Reith C (2011). The effects of lowering LDL cholesterol with simvastatin plus ezetimibe in patients with chronic kidney disease (Study of Heart and Renal Protection): a randomised placebo-controlled trial. Lancet.

[7] Vaziri ND, Norris KC (2013). Reasons for the lack of salutary effects of cholesterol-lowering interventions in end-stage renal disease populations. Blood Purif.

[8] Zamiah SA, Draman CR, Seman MR, Safhan AF, Rozalina R, Nik Ruzni NI (2018). The cardiovascular risk factor profiles among end-stage renal failure patients treated with continuous ambulatory peritoneal dialysis and intermittent hemodialysis. Saudi J Kidney Dis Transpl.

[9] Moradi H, Streja E, Vaziri ND (2018). ESRD-induced dyslipidemia-should management of lipid disorders differ in dialysis patients?. Semin Dial.

[10] Wei SS, Paganini EP, Cressman MD, Wright E (1994). Use of hemodiafiltration to enhance delivered dialysis. ASAIO J.

[11] Ottosson P, Attman PO, Knight C, Samuelsson O, Weiss L, Alaupovic P (2001). Do high-flux dialysis membranes affect renal dyslipidemia?. ASAIO J.

[12] Akoglu H, Dede F, Piskinpasa S, Falay MY, Odabas AR (2013). Impact of low- or high-flux haemodialysis and online haemodiafiltration on inflammatory markers and lipid profile in chronic haemodialysis patients. Blood Purif.

[13] Fellström BC, Jardine AG, Schmieder RE (2009). Rosuvastatin and cardiovascular events in patients undergoing hemodialysis. N Engl J Med.

[14] Solbu MD, Mjøen G, Mark PB (2018). Predictors of atherosclerotic events in patients on haemodialysis: post hoc analyses from the AURORA study. Nephrol Dial Transplant.

[15] Nemerovski CW, Lekura J, Cefaretti M, Mehta PT, Moore CL (2013). Safety and efficacy of statins in patients with end-stage renal disease. Ann Pharmacother.

[16] Lokesh S, Kadavanu TM, Green SR (2016). A comparative study of lipid profile and cardiovascular risk biomarkers among chronic haemodialysis patients and healthy individuals. J Clin Diagn Res.

[17] Eckart A, Struja T, Kutz A (2020). Relationship of nutritional status, inflammation, and serum albumin levels during acute illness: a prospective study. Am J Med.

[18] Marsen TA, Beer J, Mann H (2017). Intradialytic parenteral nutrition in maintenance hemodialysis patients suffering from protein-energy wasting. Results of a multicenter, open, prospective, randomized trial. Clin Nutr.

[19] Chertow GM, Burke SK, Raggi P (2002). Sevelamer attenuates the progression of coronary and aortic calcification in hemodialysis patients. Kidney Int.

[20] Parhofer KG (2015). Interaction between glucose and lipid metabolism: more than diabetic dyslipidemia. Diabetes Metab J.

[21] Wanner C, Krane V, März W, Olschewski M, Mann JF, Ruf G, Ritz E (2005). Atorvastatin in patients with type 2 diabetes mellitus undergoing hemodialysis. N Engl J Med.

[22] Righetti M, Wiley MH, Murrill PA, Siperstein MD (1976). The in vitro metabolism of mevalonate by sterol and non-sterol pathways. J Biol Chem.

[23] Pedersen TR, Kjekshus J, Berg K (2004). Randomised trial of cholesterol lowering in 4444 patients with coronary heart disease: the Scandinavian Simvastatin Survival Study (4S). 1994. Atheroscler Suppl.

[24] Cannon CP, Braunwald E, McCabe CH (2004). Intensive versus moderate lipid lowering with statins after acute coronary syndromes. N Engl J Med.

